# Nondestructive classification of saffron using color and textural analysis

**DOI:** 10.1002/fsn3.1478

**Published:** 2020-02-27

**Authors:** Morteza Mohamadzadeh Moghadam, Masoud Taghizadeh, Hassan Sadrnia, Hamid Reza Pourreza

**Affiliations:** ^1^ Department of Food Science and Technology Faculty of Agriculture Ferdowsi University of Mashhad Mashhad Iran; ^2^ Department of Biosystems Engineering Faculty of Agriculture Ferdowsi University of Mashhad Mashhad Iran; ^3^ Department of Computer Engineering Faculty of Engineering Ferdowsi University of Mashhad Mashhad, Iran

**Keywords:** classification, image processing, saffron

## Abstract

Saffron classification based on machine vision techniques as well as the expert's opinion is an objective and nondestructive method that can increase the accuracy of this process in real applications. The experts in Iran classify saffron into three classes Pushal, Negin, and Sargol based on apparent characteristics. Four hundred and forty color images from saffron for the three different classes were acquired, using a mobile phone camera. Twenty‐one color features and 99 textural features were extracted using image analysis. Twenty‐two classifiers were employed for classification using mentioned features. The support vector machine and Ensemble classifiers were better than other classifiers. Our results showed that the mean classification accuracy was up to 83.9% using the Quadratic support vector machine and Subspace Discriminant classifier.

## Graphical abstract

Saffron classification based on machine vision techniques as well as the expert's opinion was carried out. Twenty‐one color features and ninety‐nine textural features were extracted using image analysis. The results showed that the support vector machine and Ensemble classifiers were better than other classifiers.

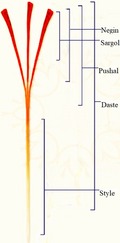

## 1 INTRODUCTION

**Figure 1:**
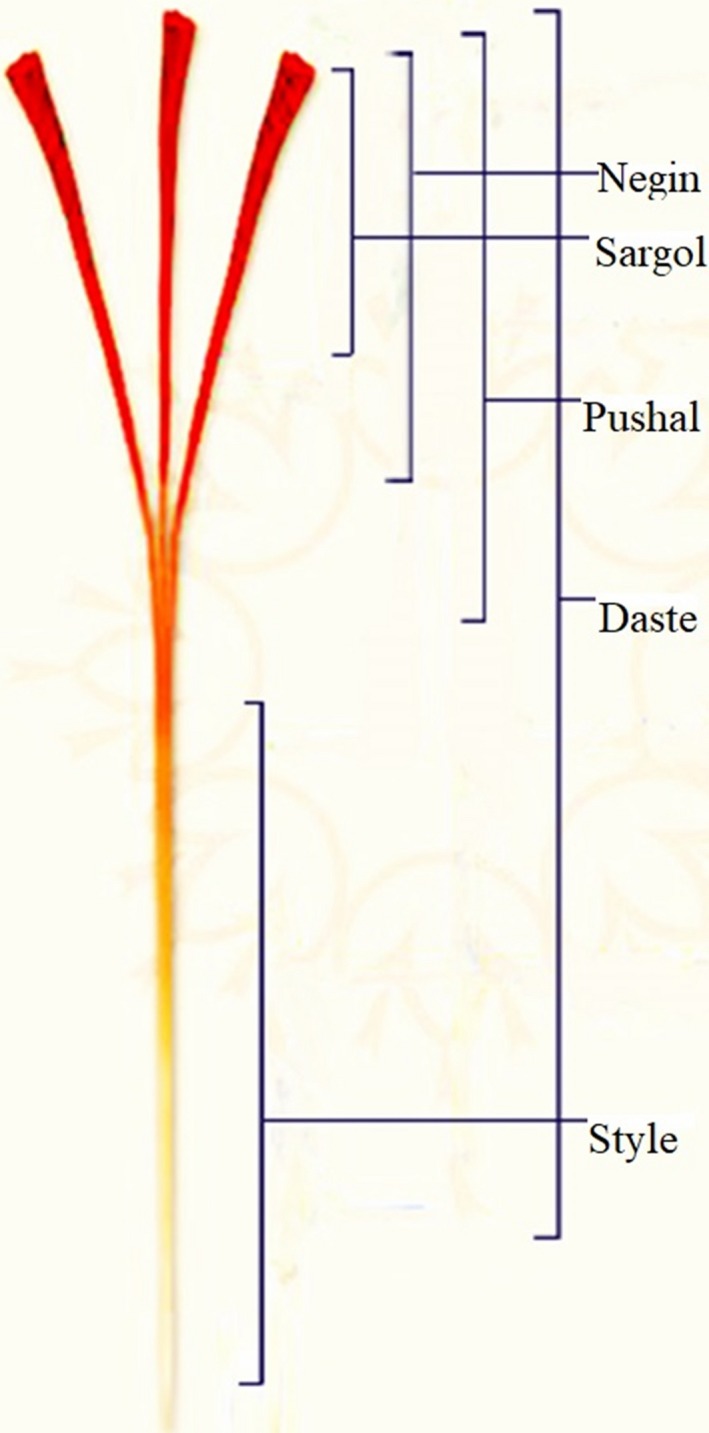
Different types of saffron including Negin, Sargol, Pushal, and Daste

Saffron is the most expensive spice in the world. This product is cultivated in different countries of the world, such as Iran, India, Spain, Greece, Italy, and Morocco (Fernández, 2004). Iran is the largest saffron producer in the world. Currently, 94% of the world's saffron is produced in Iran (Masi et al., 2016). Traditionally, saffron classification based on apparent qualitative parameters is graded by expert people (Kiani & Minaei, 2016). In different countries such as Iran, Spain, and India, pure saffron is graded according to the apparent characteristics of saffron to different categories. In the local market of Iran, saffron is divided into three types: Sargol, Negin, and Pushal (Peter, 2012) and (Shahdadi, Barati, Bahador, & Eteghadi, 2016). There is another type of saffron in Iran that is called Daste or Dokhtarpich or Bunch (red stigmas plus large amounts of yellow style, presented in a tiny bundle) (Bonyadi, Yazdani, & Saadat, 2014). This type is less available on the market in Iran and is not traded Internationally (Azarabadi & Özdemir, 2018). To prepare Pushal, the stigmas (section of the aerial part of the pistil) are separated from the ends of the three filaments with a small amount of style (part of the pistil between the stigma and the ovary) and then dried. Negin is only red‐colored; three filaments separated and collected individually and then dried. The difference between Sargol and Negin is that the stigma has been broken, but in Negin, mostly stigmas are whole and without crushed filaments (Figure 1) (Atefi, Akbari Oghaz, & Mehri, 2013) and (Kafi, Koocheki, & Rashed, 2006). Sargol saffron consists of only the red part of the stigma. This category of saffron has a strong coloring property (Azarabadi & Özdemir, 2018). Due to the difference between expert opinions, there are errors that can be avoided using an objective approach such as image processing (Pourreza, Pourreza, Abbaspour‐Fard, & Sadrnia, 2012). Advances in machine vision technology make accurate, robust, and low‐cost vision machine systems that make it suitable for detection food quality and so this technology can be used to determine the quality of saffron (Kiani & Minaei, 2016).

Kiani, Minaei, and Ghasemi‐Varnamkhasti (2018) propose the use of E‐nose, E‐tongue, and CVS systems to evaluate saffron quality and replace sensory recognition by human assessors (Kiani et al., 2018). Minaei, Kiani, Ayyari, and Ghasemi‐Varnamkhasti (2017) demonstrated that the combination of computer vision system (CVS) and multilayer perceptron (MLP) is a simple tool for evaluating the quality of saffron samples based on color strength. The performance of the MLP model for saffron color recognition was better than PLS and MLR, and the success rate of classification (CSR) was 96.67%. (Minaei et al., 2017). Today, color computer vision systems are used in various food industries and agricultural products sorting systems because they are reliable, fast, and inexpensive (Donis‐González & Guyer, 2016). Color computer vision is used to categorize or recognize the quality of agricultural products and various types of foods, including dates (Muhammad, 2015), pistachios (Omid, Firouz, Nouri‐Ahmadabadi, & Mohtasebi, 2017), apple (Paulus & Schrevens, 1999), pizza (Sun, 2016), and Wheat (Pourreza et al., 2012). The computer vision system is trained based on specific patterns extracted from a set of color images provided for different classes, such as texture, geometry, and color properties. Then, the computer vision system determines which new image belongs to which particular category (Faucitano, Huff, Teuscher, Gariepy, & Wegner, 2005). The first step involves extracting a large number of features from classified images. Then, the features must have the ability to separate the classes correctly, which, by training the system, can automatically categorize the new image. Classification is performed by statistical algorithms and different clustering by assigning each image to the corresponding class (Donis‐González & Guyer, 2016). The purpose of this study was to design a visual machine technique for detecting different types of saffron (Sargol, Negin, and Pushal) using images taken with mobile phones from bulk samples. Texture properties, color properties, and the percentage of foreign matter (based on color) of saffron were obtained.

## 2 MATERIALS AND METHODS

### 2.1 Saffron samples

A total of 440 samples of different saffron kinds on the market were prepared, without any additives, from various cities of Khorasan Province: Gonabad, Bajestan, Roshtkhar, Sabzevar, Mashhad, Torbat Heydarieh, and Kakhk, without any additives as fraud, and then, the samples were coded. Four experts who had a long history of saffron trading were selected. They divided the specimens into three classes, Sargol, Negin, and Pushal. Samples' information was recorded in a database (Zheng & Lu, 2012) and (Donis‐González & Guyer, 2016) and (Zhang, Lee, Lillywhite, & Tippetts, 2017).

### 2.2 Image acquisition

Image acquisition was done with a cellphone camera (Samsung Galaxy S7 Edge SM‐G935FD Dual SIM 32GB Mobile Phone), which was placed on an imaging chamber at a distance of 9 cm from the sample. In the lighting system, SMD LED strip lights (4014 SMD LED Module) have been used in the upper part of the imaging chamber. A diffuser was installed for the uniformity of light under the lamps. The black background color was used to create the best contrast. The shutter speed was 1/500 s without employing flash, and, respectively, lens focal length, Diaphragm value, and ISO were 4/2 mm, F1/7, and 100. Images were captured at their maximum resolution (3024 × 4032 pixels) and were saved in “JPG” format. For imaging, the images were transferred to the laptop, which was equipped with MATLAB software (2017b. ver. 9.3). The images were given to the expert individuals to classify the samples into three classes: Sargol, Negin, and Pushal. Based on the average view of the experts, 440 different samples were taken from them, and they were divided into three categories: 195 samples: Pushal, 129 samples: Negin, and 116 samples: Sargol. In this case, the average views of the experts were selected as the criteria for tagging the samples.

### 2.3 Image preprocessing

**Figure 2:**
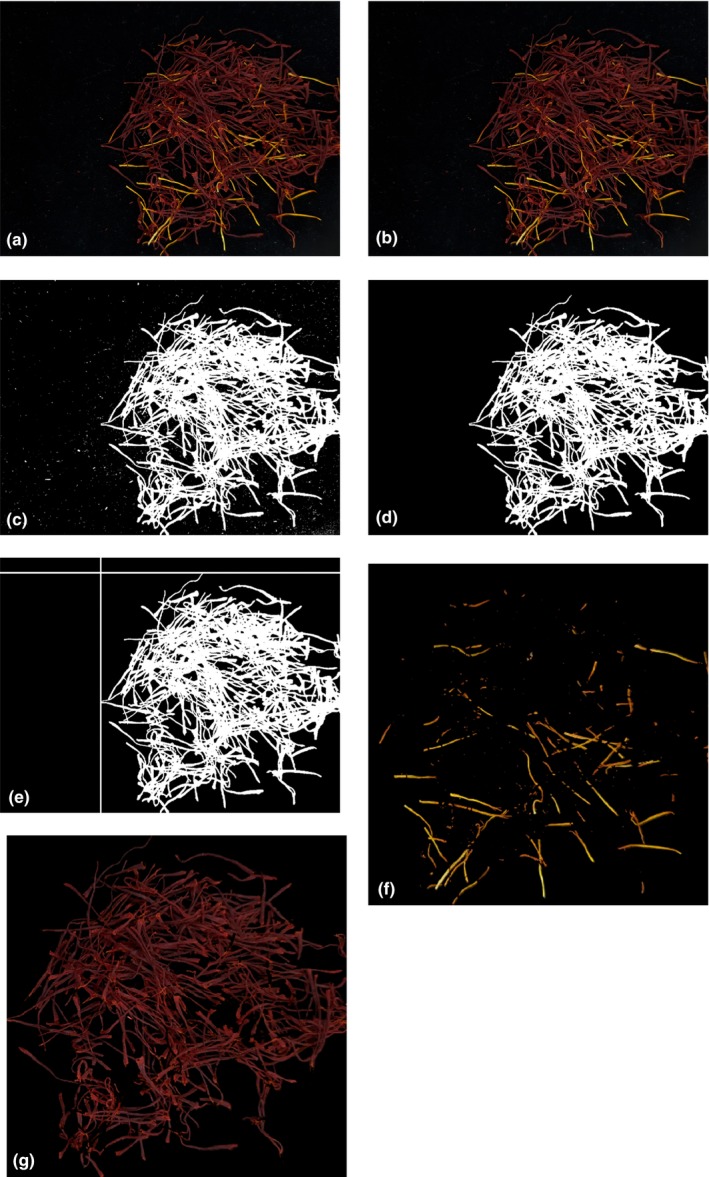
Image preprocessing: (a) original sample image, (b) smoothed image, (c) foreground of the image,(d) binary image, (e) selecting cropped area, (f) yellow and white parts, and (g) Pure saffron parts

Original sample image is presented in Figure 2a. In the first step, in order to remove the noises and smooth it, the image is filtered using a low‐pass filter. The result is shown in Figure 2b. Foreground of the image is selected by choosing the pixels having intensity bigger than 20. Results are shown in Figure 2c. Small objects are removed from foreground binary image by morphological opening operation the image where all connected components (objects) that have fewer than 3,000 pixels are removed. Further, the image is eroded and dilated by a morphological structuring element with 5‐pixel radius. The final foreground of the image is shown in Figure 2d. The saffron part of image is cropped by selecting the area, which has nonzero values. For this purpose, the projection of image over vertical and horizontal axis is calculated and the area between minimum and maximum values is cropped. For example, for the sample image, the area between two vertical and horizontal lines shown in Figure 2e is selected. In general, four virtual lines are generated for defining the cropped area. The cropped area image is then used for further processing.

### 2.4 Color image feature extraction

Color components were extracted from color images of each saffron sample. The components of various color spaces including R, G, B, H, S, r, L*, a*, b*, C, I, E, Y, Cb, Cr, Y, I, and Q were extracted from the images. Yellow and white parts of the original cropped image would be selected using the Color Thresholder App. The image is entered the tool in HSI format. The proper threshold for hue value is selected by visual inspecting. Minimum and maximum values for hue in this case are 0.045, and 0.279, respectively. Figure 2f and Figure 2g is showing the result of applying these thresholds. At the end, the percentage of foreign matters (yellow and white parts based on pixels) in the total mass, the percentage of stigmas (red parts based on pixels) in the total mass, the proportion of foreign matters to the stigmas based on percentage, and the components of various color spaces were extracted from the images.

### 2.5 Textural algorithm

Texture analysis is one of the most important characteristics used in identifying regions of interest in an image and has been widely used in image processing. They are defined as attributes representing spatial arrangement of the gray levels of pixels in a region of a digital image, which provide measures of some properties of a region such as smoothness, coarseness, and regularity (Wang, Zhang, & Wei, 2019). To analyze the textures, the features extracted from the image are local entropy of grayscale image (entropy), local standard deviation of image (STD), local binary patterns (LBP), and gray level co‐occurrence matrix (GLCM). Features extracted from GLCM include contrast, homogeneity, correlation, and energy that the mentioned features were extracted from the images. The contrast shows the intensity of the gray variation in the image.

(1) $$\text{Contrast}=\sum_{i,j}{\left|i-j\right|}^{2}p\left(i.j\right)$$

The correlation describes the linearity and dependence of a different two‐pixel value. In this case, *μ* is the mean value of the matrix and *σ_i_σ_j_* of the variance.

(2) $$\text{Correlation}=\sum_{i.j}\frac{\left(i-\mu j\right)\left(j-\mu j\right)p\left(i.j\right)}{\sigma_{i}\sigma_{j}}$$

The energy represents the order of the image (repetition of the pixel pair) and in fact represents the smoothness and uniformity of the sample surface.

(3) $$\text{Energy}=\sum_{i.j}p{\left(i.j\right)}^{2}$$

Homogeneity describes the similarity of a pixel with neighboring pixels and reflects the uniformity of the image.

(4) $$\text{Homogeneity}=\sum_{i.j}\frac{p\left(i.j\right)}{1+\left|\left(i-j\right)\right|}$$

Specifications extracted from entropy, standard deviation, and local binary patterns were calculated according to Table 1. In addition, the histogram is a graphical representation of the number of pixels for each brightness level in the input image. We defined 25 Bin in this study, and in each period, the abundance of things was gathered together and placed there. Finally, 120 features were extracted from each image.

**Table 1:** Features extracted from entropy, standard deviation, and local binary patterns matrices

Feature	Equation
Mean	$\mu =\sum_{i}p\left(i\right)$
Standard deviation	$\sigma =\sqrt{\sum_{i}{\left(i-\mu \right)}^{2}p\left(i\right)}$
Smoothness	$1-\frac{1}{\left(1+\sigma^{2}\right)}$
Third moment	$\sum_{i}{\left(i-\mu \right)}^{3}p\left(i\right)$
Uniformity	$\sum_{i}p{\left(i\right)}^{2}$
Entropy	$-\sum_{i}p\left(i\right)\log\left\{\left(i\right)\right\}$
Gray level rage	$\max\left\{\text{i|p}\left(i\right)\neq 0\right\}-\min\left\{\text{i|p}\left(i\right)\neq 0\right\}$

#### 2.5.1 The local binary patterns (LBPs)

A local binary pattern is a synergistic approach to texture analysis, which can provide a boundary of proximity with a pixel tag and a binary result. The main advantage of LBP in business applications is its ability to maintain independent behavior with grayscale level changes and its computational efficiency, processing images in complex real‐time environment. In a basic LBP, each 3 × 3 neighborhood is thresholded by the value of the central pixel. Then, the threshold neighborhood values are multiplied by weights given to the corresponding pixels. Finally, the resulted values are summed to acquire the number of this texture unit (Pantazi, Moshou, & Tamouridou, 2019).

### 2.6 Classification model

The features outlined in the above sections were used to classify. 22 different calssifiers were used including:

#### 2.6.1 Decision trees classifiers

Decision tree (DT) is a machine learning algorithm which classifies the training data recursively by each node in order to maximize the separation of data. The decisions in the tree are started from the root node down to a leaf node to predict a response. The leaf node contains the response (Kamiński, Jakubczyk, & Szufel, 2018). Types of models used in this group include Fine Tree, Medium Tree, and Coarse Tree.

#### 2.6.2 Discriminant analysis classifiers

Discriminant analysis is a classification method. It is a multivariate classification technique. It assumes that different classes generate data based on different Gaussian distributions (Riveiro‐Valiño, Álvarez‐López, & Marey‐Pérez, 2009). Types of models used in this group include linear discriminant analysis and quadratic discriminant analysis.

#### 2.6.3 Support vector machine classifiers

Support vector machine (SVM) is an effective modeling tool for classification and was used for regression, pattern classification, prediction, and problem detection (Nasirahmadi et al., 2019). In SVM, data input space is mapped into a high dimensional feature space through a kernel function by using minimal training data (Huang, Tang, Yang, & Zhu, 2016). Types of models used in this group include Linear SVM, Quadratic SVM, Cubic SVM, Fine Gaussian SVM, Medium Gaussian SVM, and Coarse Gaussian SVM.

#### 2.6.4 Nearest neighbor classifiers

The Nearest neighbor classifiers in the low‐precision dimensions is a good predictor. However, they may not have this capability on a large scale. In this classifier, samples that are neighbors or similar to a well‐known instance are identified that fall into the set of training, and then, the classification is done based on the training set (Xie, Yang, & He, 2017). Types of models used in this group include Fine KNN, Medium KNN, Coarse KNN, Cosine KNN, Cubic KNN, and Weighted KNN.

#### 2.6.5 Ensemble classifiers

An ensemble is a supervised learning approach such as bagging, boosting, and variants that use multiple models to improve the predictive performance than could be obtained from any of the constituent models (Dutta et al., 2015). Types of models used in this group include Boosted Trees, Bagged Trees, Subspace Discriminant, Subspace KNN, and RUSBoost Trees**.**

### 2.7 Validation and performance evaluation indices

A fivefold stratified cross‐validation technique was used to validate the classification. In k‐fold cross‐validation, the original sample is randomly divided into k equal sized subsamples. Of the k subsamples, a single subsample is remained as the validation data for testing the model, and the remaining/k subsamples are used as training data. The cross‐validation process is then repeated k times, with each of the k subsamples used exactly once as the validation data. The k results can then be averaged to produce a single estimation. The advantage of this method over repeated random subsampling is that all observations are used for both training and validation, and each observation is used for validation exactly once (Siedliska, Baranowski, & Mazurek, 2014). Accuracy, confusion matrix, true‐positive rate (TP rate), false‐negative rate (FN rate), positive predictive rate (PP rate), and false discovery rate (FD rate) were calculated (Xie et al., 2017). Also, the receiver operating characteristic (ROC) was computed in MATLAB based on true‐positive and false‐negative rates. The area under the ROC curve which ranges from 0.5 (no discrimination ability) to 1 (best discrimination ability) was also calculated (Nasirahmadi et al., 2019).

One‐way analysis of variance (ANOVA) and Duncan's test were used to determine the significant difference between the accuracy of classifiers. Statistical analysis was performed using SPSS software (IBM Statistics version 23).

## 3 RESULTS AND DISCUSSION

The 440 color photographs from different samples of saffron including 195 samples of Pushal, 129 Negin, and 116 Sargol were used in this study. The glossary defined for classifiers, including 21 color features and 99 texture features, was extracted from 440 samples. The classifier was then evaluated using fivefold cross‐validation. In the cross‐validation, the original samples were randomly partitioned into five groups. Four groups were used as training data for developing the model, and the remaining group was retained as validation data for testing the classifier. The process was repeated for five times, with each of the groups used once as the validation data (Kuo, Chung, Chen, Lin, & Kuo, 2016).

### 3.1 Classification when features of color were used in the classifiers

Table 2 illustrates the accuracy of 22 classifiers using 21 color features. Based on the results, it can be seen that classification with Linear Discriminant, Linear SVM, Bagged Trees, and RUSBoost Trees classifiers have higher average accuracy compared other classifiers. The average accuracy of these four classifiers did not differ significantly (*p* < .05). For Linear SVM classifier, the classification accuracy was 82.23% (±0.66%).

**Table 2:** Average classification accuracies (%) for 10 times running of fivefold cross‐validation using 21 color features for saffron classification

NO.	Classifier	Average accuracy %	*SD* %	NO.	Classifier	Average accuracy %	*SD* %
1	Fine Tree	79.65	1.43	12	Fine KNN	77.33	1.26
2	Medium Tree	80.86	1.68	13	Medium KNN	77.71	0.81
3	Coarse Tree	79.58	0.77	14	Coarse KNN	73.5	0.4
4	Linear Discriminant	82.23	0.66	15	Cosine KNN	77.69	1.18
5	Quadratic Discriminant	58.17	0.66	16	Cubic KNN	78.02	0.87
6	Linear SVM	82.27	0.7	17	Weighted KNN	79.39	0.85
7	Quadratic SVM	80.73	0.69	18	Boosted Trees	81.09	0.97
8	Cubic SVM	78.89	0.8	19	Bagged Trees	82.18	1.04
9	Fine Gaussian SVM	79.34	0.68	20	Subspace Discriminant	80.65	0.55
10	Medium Gaussian SVM	81.11	0.58	21	Subspace KNN	60.71	3.12
11	Coarse Gaussian SVM	76.7	0.39	22	RUSBoost Trees	81.83	1.19

### 3.2 Classification when features of texture were used in the classifiers

Table 3 illustrates the accuracy of 22 classifiers using 99 texture features. The results showed that the average classification accuracy of Subspace Discriminant Classifier was higher than the other classifiers, with a significant difference (*p* < .05). The classification accuracy of the Subspace Discriminant Classifier was 82.83% (±0.85%).

**Table 3:** Average classification accuracies (%) for 10 times running of fivefold cross‐validation using 99 texture features for saffron classification

NO.	Classifier	Average accuracy %	*SD* %	NO.	Classifier	Average accuracy %	*SD* %
1	Fine Tree	69.93	2	12	Fine KNN	75.99	1.08
2	Medium Tree	72.16	1.56	13	Medium KNN	75.55	0.93
3	Coarse Tree	71.42	0.82	14	Coarse KNN	74.82	0.85
4	Linear Discriminant	72.26	0.91	15	Cosine KNN	76.6	0.98
5	Quadratic Discriminant	44.3	0	16	Cubic KNN	76.09	0.79
6	Linear SVM	80.3	0.69	17	Weighted KNN	77.75	0.68
7	Quadratic SVM	79.53	1.16	18	Boosted Trees	76.76	1.05
8	Cubic SVM	78.67	1.41	19	Bagged Trees	77.61	1.81
9	Fine Gaussian SVM	78.02	1.05	20	Subspace Discriminant	82.83	0.85
10	Medium Gaussian SVM	79.06	0.97	21	Subspace KNN	46.12	1.55
11	Coarse Gaussian SVM	74.01	0.83	22	RUSBoost Trees	75.94	1.21

### 3.3 Classification when combinations of all features were used in the classifier

Table 4 shows the average accuracy of the classifiers for classifying saffron into three classes of Sargol, Negin, and Pushal. The ANOVA and Duncan's test showed that the average accuracy of classifiers Linear SVM (LSVM), Quadratic SVM (QSVM), Cubic SVM (CSVM), Medium Gaussian SVM (MGSVM), Boosted Trees (BoT), Bagged Trees (BaT), and Subspace Discriminant (SDT) did not differ significantly (*p* < .05). The results of this study show that seven classifiers mentioned are qualified to separate the saffron to three classes Sargol, Negin, and Pushal from others. It was also found that SVM and Ensemble Classifiers were better than other classifiers for classification of saffron. Based on the results, it can be seen that classification with all features resulted in higher average classification accuracy compared to Color and texture features separately. For Quadratic SVM classifier, the average accuracy was 83.9% (±0.69%), and the classification accuracy of Subspace Discriminant classifier was obtained 83.9% (±0.36%.). The results show that the accuracy of saffron category identification can increase when color features are used in combination with textural features.

**Table 4:** Average classification accuracies (%) for 10 times running of fivefold cross‐validation using 120 color and texture features for saffron classification

NO.	Classifier	Average accuracy %	*SD* %	NO.	Classifier	Average accuracy %	*SD* %
1	Fine Tree	80	1.33	12	Fine KNN	79.1	0.46
2	Medium Tree	80.5	1.18	13	Medium KNN	80.5	0.46
3	Coarse Tree	78	0.92	14	Coarse KNN	77.05	0.87
4	Linear Discriminant	72.3	0.93	15	Cosine KNN	80.2	0.64
5	Quadratic Discriminant	44.3	0	16	Cubic KNN	81.4	0.44
6	Linear SVM	83.1	0.83	17	Weighted KNN	81.4	0.37
7	Quadratic SVM	83.9	0.69	18	Boosted Trees	83.85	0.57
8	Cubic SVM	82.7	1.06	19	Bagged Trees	83.3	1.29
9	Fine Gaussian SVM	79.3	0.69	20	Subspace Discriminant	83.9	0.36
10	Medium Gaussian SVM	83.5	0.47	21	Subspace KNN	45.8	0.71
11	Coarse Gaussian SVM	77.5%	0.54	22	RUSBoost Trees	81.75	0.71

**Figure 3:**
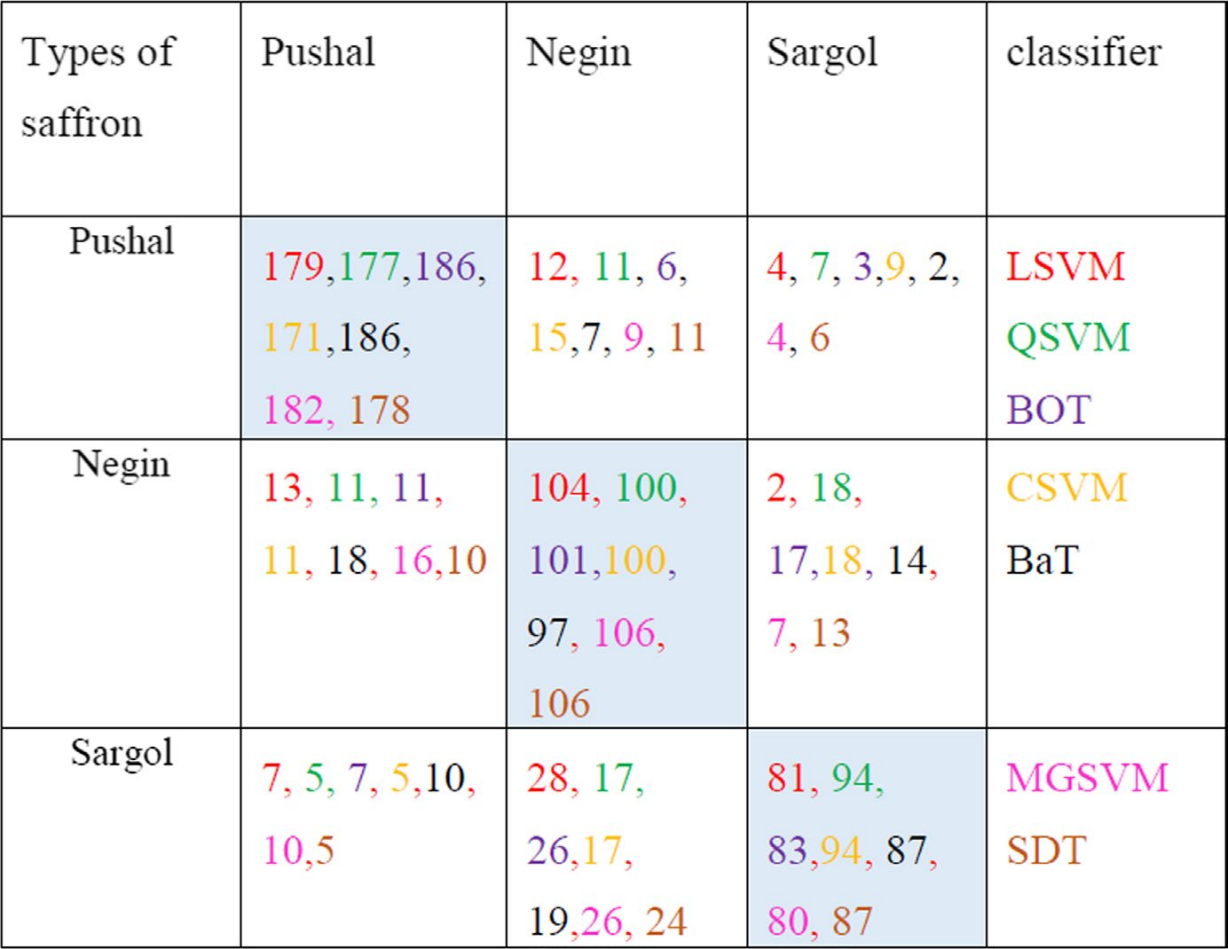
Confusion matrices of seven classifiers for distinguishing Pushal, Negin, and Sargol samples (Confusion matrices of the classification models for cultivars as an independent variable). Each model has a specific color representation and the diagonal cells (in blue) present the correct classifications

Figure 3 shows the confusion matrix for seven classifiers mentioned. Also, detailed accuracy analysis has been reported in Table 5. A high value of TP rate and PP rate, and a low value of FN rate and FD rate, mean the classification model is good. These values for Pushal saffron were better than other classes of saffron. The FN rate and FD rate showed that the classification error of Sargol and Negin is more than Pushal. These errors happen when the values are close to each other, and it is hard to classify them. In terms of appearance, Negin and Sargol are very similar, and the distinction between them is difficult. In the Pushal, three filaments of stigmas are connected, which at the end has a bit of style, but in the Negin and Sargol, three filaments of stigmas are separated.

**Table 5:** Detailed accuracy analysis using class of the studied seven classifiers

Classification model	TP rate (Pushal) %	TP rate (Negin) %	TP rate (Sargol) %	FN rate (Pushal) %	FN rate (Negin) %	FN rate (Sargol) %	PP rate (Pushal) %	PP rate (Negin) %	PP rate (Sargol) %	FD rate (Pushal) %	FD rate (Negin) %	FD rate (Sargol) %
LSVM	92	81	70	8	19	30	90	72	84	10	28	16
QSVM	91	78	81	9	22	19	92	78	79	8	22	21
CSVM	88	78	81	12	22	19	91	76	78	9	24	22
MGSVM	93	82	69	7	18	31	88	75	88	13	25	12
BOT	95	78	72	5	22	28	91	76	81	9	24	19
BaT	95	75	75	5	25	25	87	79	84	13	21	16
SDT	91	82	75	9	18	25	92	75	82	8	25	18

**Figure 4:**
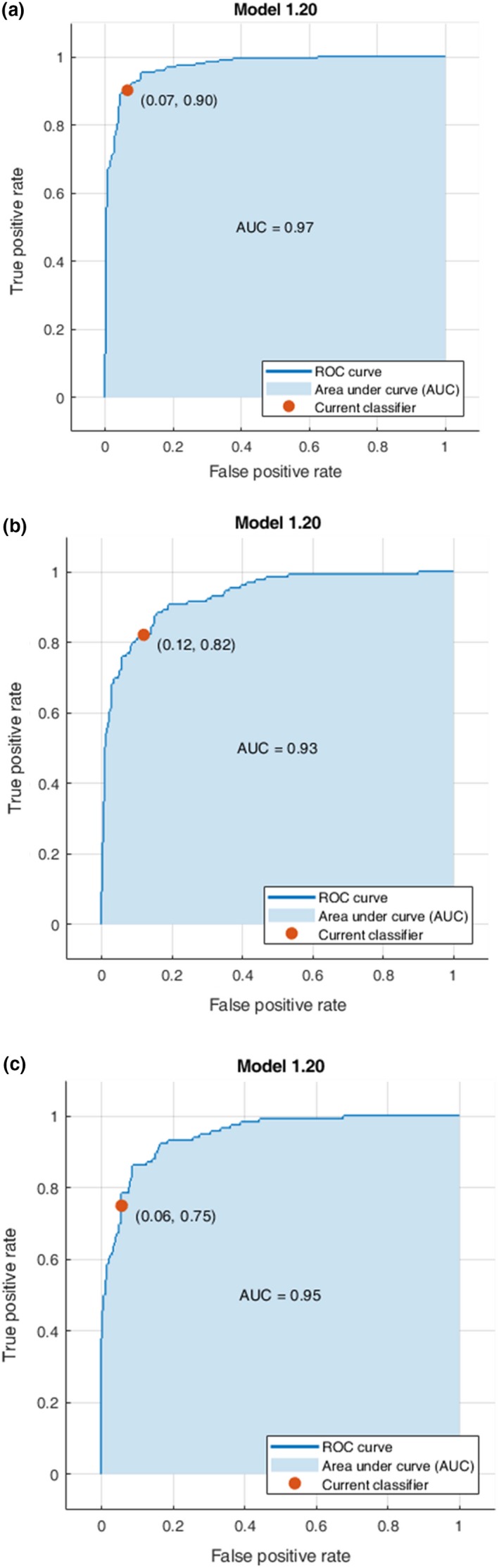
The ROC along with AUC values of the Subspace Discriminant classifier: (a) Pushal, (b) Negin, and (c) Sargol

The receiver operating characteristics (ROC) was an additional method for evaluating the performance of the classification models. An ROC graph illustrates relative trade‐offs between true‐positives and false‐positives and its x‐axis is the false‐positive rate, whereas the y‐axis is the true‐positive rate of the model (Siedliska et al., 2014). The area under the ROC curve (AUC) is an important statistical parameter for evaluating classifier performance. Figure 4 shows the ROC curves, along with the AUC, for each class Pushal, Negin, and Sargol obtaining of the Subspace Discriminant classifier. The AUC values obtained were 0.96 for Pushal, 0.91 for Negin, and 0.93 for Sargol. The closer AUC is to 1, the better overall diagnostic performance of established classifier (Hu, Dong, & Liu, 2016). In Table 6, the results show that AUC values for Pushal were better than other classes of saffron. The SDT had the highest AUC values for identifying Pushal, Negin, and Sargol classes. Moreover, the overall AUC values of the classification LSVM, QSVM, CSVM, MGSVM, BoT, BaT, and SDT methods were 0.936, 0.933, 0.916, 0.936, 0.930, 0.943, and 0.95, respectively. These results further revealed that in this study Subspace Discriminant classifier had a success in the classification of saffron classes using the textural features and the combination features. According to the results of Tables 5 and 6, the classification of Pushal saffron with these models was better than the other two classes.

**Table 6:** The AUC (area under the ROC curve) of the seven classifiers for different saffron classes

Classification model	Pushal	Negin	Sargol	Overall
LSVM	0.97	0.91	0.93	0.936
QSVM	0.96	0.91	0.93	0.933
CSVM	0.95	0.90	0.90	0.916
MGSVM	0.96	0.91	0.94	0.936
BOT	0.96	0.90	0.93	0.93
BaT	0.97	0.92	0.94	0.943
SDT	0.97	0.93	0.95	0.95

Results from this study show that color images, obtained using a mobile phone camera, were ideal for this experiment. This technique classifies saffron into three classes by measuring different color, and textural features from color images. The Local traders in Iran considering the type of saffron (Pushal, Negin, and sargol) determine the price, because this classification has a significant relationship with the quality of saffron (Azarabadi & Özdemir, 2018). Few studies have been done on the classification of saffron using machine learning methods. The method described in this study can be a valuable tool for increasing the accuracy of pricing and assurance for the customer to purchase the product. Using other methods of machine learning and morphological characteristics of saffron can improve the technique used in this study. Further studies can lead to the creation of application software that can be used by the end user for classification of saffron.

## 4 CONCLUSIONS

In summary, these results showed that the visual texture and color index could be a good index for separating saffron of Pushal, Negin, and Sargol. The saffron samples were collected from the cities of Khorasan Province. A commercially available mobile phone was used to capture the saffron images. The images were given to expert individuals to classify the samples into three classes: Sargol, Negin, and Pushal. A total number of 120 features were extracted from the saffron images. Textures and color features were considered as inputs to 22 different classifiers for classification of saffron. The SVM and Ensemble Classifiers were better than other Classifiers. The classification accuracy 83.9% was achieved from the Quadratic SVM classifier and Subspace Discriminant classifier. Future studies on morphology features and machine learning techniques (i.e. deep learning) can optimize the accuracy of saffron class identification.

## CONFLICT OF INTEREST

None declared.

## AUTHORS' CONTRIBUTIONS

The first author was responsible for the accomplishment of most of the works, searching literature data, and write up of the paper. The second author also contributed in the manuscript preparation and standardized the paper as well as supervision of the whole research works. The third and fourth authors also contributed in the manuscript preparation. All authors approved the final manuscript for publication.

## ETHICAL STATEMENT

This study does not involve any human or animal testing.

## References

[fsn31478-bib-0001] Atefi, M. , Akbari Oghaz, A. R. , & Mehri, A. (2013). Drying effects on chemical and sensorial characteristics of saffron. Iranian Journal of Nutrition Sciences & Food Technology, 8(3), 201–208.

[fsn31478-bib-0002] Azarabadi, N. , & Özdemir, F. (2018). Determination of crocin content and volatile components in different qualities of Iranian saffron. GIDA/The Journal of FOOD, 43(3), 476–489. 10.15237/gida.GD18018

[fsn31478-bib-0003] Donis‐González, I. R. , & Guyer, D. E. (2016). Classification of processing asparagus sections using color images. Computers and Electronics in Agriculture, 127, 236–241. 10.1016/j.compag.2016.06.018

[fsn31478-bib-0004] Dutta, R. , Smith, D. , Rawnsley, R. , Bishop‐Hurley, G. , Hills, J. , Timms, G. , & Henry, D. (2015). Dynamic cattle behavioural classification using supervised ensemble classifiers. Computers and Electronics in Agriculture, 111, 18–28. 10.1016/j.compag.2014.12.002

[fsn31478-bib-0005] Faucitano, L. , Huff, P. , Teuscher, F. , Gariepy, C. , & Wegner, J. (2005). Application of computer image analysis to measure pork marbling characteristics. Meat Science, 69(3), 537–543. 10.1016/j.meatsci.2004.09.010 22062993

[fsn31478-bib-0006] Fernández, J.‐A. (2004). Biology, biotechnology and biomedicine of saffron. Recent Research Developments in Plant Science, 2, 127–159.

[fsn31478-bib-0007] Hu, M.‐H. , Dong, Q.‐L. , & Liu, B.‐L. (2016). Classification and characterization of blueberry mechanical damage with time evolution using reflectance, transmittance and interactance imaging spectroscopy. Computers and Electronics in Agriculture, 122, 19–28. 10.1016/j.compag.2016.01.015

[fsn31478-bib-0008] Huang, M. , Tang, J. , Yang, B. , & Zhu, Q. (2016). Classification of maize seeds of different years based on hyperspectral imaging and model updating. Computers and Electronics in Agriculture, 122, 139–145. 10.1016/j.compag.2016.01.029

[fsn31478-bib-0009] Jabbarpoor Bonyadi, M. H. , Yazdani, S. , & Saadat, S. (2014). The ocular hypotensive effect of saffron extract in primary open angle glaucoma: A pilot study. BMC Complementary and Alternative Medicine, 14(1), 399 10.1186/1472-6882-14-399 25319729 PMC4213480

[fsn31478-bib-0010] Kafi, M. , Koocheki, A. , & Rashed, M. H. (2006). Saffron (Crocus sativus): Production and processing. Enfield, NH, USA: Science Publishers.

[fsn31478-bib-0011] Kamiński, B. , Jakubczyk, M. , & Szufel, P. (2018). A framework for sensitivity analysis of decision trees. Central European Journal of Operations Research, 26(1), 135–159. 10.1007/s10100-017-0479-6 29375266 PMC5767274

[fsn31478-bib-0012] Kiani, S. , & Minaei, S. (2016). Potential application of machine vision technology to saffron (*Crocus * *sativus* L.) quality characterization. Food Chemistry, 212, 392–394. 10.1016/j.foodchem.2016.04.132 27374547

[fsn31478-bib-0013] Kiani, S. , Minaei, S. , & Ghasemi‐Varnamkhasti, M. (2018). Instrumental approaches and innovative systems for saffron quality assessment. Journal of Food Engineering, 216, 1–10. 10.1016/j.jfoodeng.2017.06.022

[fsn31478-bib-0014] Kuo, T.‐Y. , Chung, C.‐L. , Chen, S.‐Y. , Lin, H.‐A. , & Kuo, Y.‐F. (2016). Identifying rice grains using image analysis and sparse‐representation‐based classification. Computers and Electronics in Agriculture, 127, 716–725. 10.1016/j.compag.2016.07.020

[fsn31478-bib-0015] Masi, E. , Taiti, C. , Heimler, D. , Vignolini, P. , Romani, A. , & Mancuso, S. (2016). PTR‐TOF‐MS and HPLC analysis in the characterization of saffron (*Crocus * *sativus* L.) from Italy and Iran. Food Chemistry, 192, 75–81. 10.1016/j.foodchem.2015.06.090 26304322

[fsn31478-bib-0016] Minaei, S. , Kiani, S. , Ayyari, M. , & Ghasemi‐Varnamkhasti, M. (2017). A portable computer‐vision‐based expert system for saffron color quality characterization. Journal of Applied Research on Medicinal and Aromatic Plants, 7, 124–130. 10.1016/j.jarmap.2017.07.004

[fsn31478-bib-0017] Muhammad, G. (2015). Date fruits classification using texture descriptors and shape‐size features. Engineering Applications of Artificial Intelligence, 37, 361–367. 10.1016/j.engappai.2014.10.001

[fsn31478-bib-0018] Nasirahmadi, A. , Sturm, B. , Olsson, A.‐C. , Jeppsson, K.‐H. , Müller, S. , Edwards, S. , & Hensel, O. (2019). Automatic scoring of lateral and sternal lying posture in grouped pigs using image processing and support vector machine. Computers and Electronics in Agriculture, 156, 475–481. 10.1016/j.compag.2018.12.009

[fsn31478-bib-0019] Omid, M. , Firouz, M. S. , Nouri‐Ahmadabadi, H. , & Mohtasebi, S. S. (2017). Classification of peeled pistachio kernels using computer vision and color features. Engineering in Agriculture, Environment and Food, 10(4), 259–265. 10.1016/j.eaef.2017.04.002

[fsn31478-bib-0020] Pantazi, X. E. , Moshou, D. , & Tamouridou, A. A. (2019). Automated leaf disease detection in different crop species through image features analysis and one class classifiers. Computers and Electronics in Agriculture, 156, 96–104. 10.1016/j.compag.2018.11.005

[fsn31478-bib-0021] Paulus, I. , & Schrevens, E. (1999). Shape characterization of new apple cultivars by Fourier expansion of digitized images. Journal of Agricultural Engineering Research, 72(2), 113–118. 10.1006/jaer.1998.0352

[fsn31478-bib-0022] Peter, K. V. (2012). Handbook of herbs and spices. Amsterdam, The Netherlands: Elsevier.

[fsn31478-bib-0023] Pourreza, A. , Pourreza, H. , Abbaspour‐Fard, M.‐H. , & Sadrnia, H. (2012). Identification of nine Iranian wheat seed varieties by textural analysis with image processing. Computers and Electronics in Agriculture, 83, 102–108. 10.1016/j.compag.2012.02.005

[fsn31478-bib-0024] Riveiro‐Valiño, J. A. , Álvarez‐López, C. J. , & Marey‐Pérez, M. F. (2009). The use of discriminant analysis to validate a methodology for classifying farms based on a combinatorial algorithm. Computers and Electronics in Agriculture, 66(2), 113–120. 10.1016/j.compag.2008.12.001

[fsn31478-bib-0025] Shahdadi, H. , Barati, F. , Bahador, R. S. , & Eteghadi, A. (2016). Clinical applications of saffron (*Crocus * *sativus*) and its constituents: A literature review. Der Pharmacia Lettre, 8(19), 205–209.

[fsn31478-bib-0026] Siedliska, A. , Baranowski, P. , & Mazurek, W. (2014). Classification models of bruise and cultivar detection on the basis of hyperspectral imaging data. Computers and Electronics in Agriculture, 106, 66–74. 10.1016/j.compag.2014.05.012

[fsn31478-bib-0027] Sun, D.‐W. (2016). Computer vision technology for food quality evaluation. Cambridge, Massachusetts: Academic Press.

[fsn31478-bib-0028] Wang, A. , Zhang, W. , & Wei, X. (2019). A review on weed detection using ground‐based machine vision and image processing techniques. Computers and Electronics in Agriculture, 158, 226–240. 10.1016/j.compag.2019.02.005

[fsn31478-bib-0029] Xie, C. , Yang, C. E. , & He, Y. (2017). Hyperspectral imaging for classification of healthy and gray mold diseased tomato leaves with different infection severities. Computers and Electronics in Agriculture, 135, 154–162. 10.1016/j.compag.2016.12.015

[fsn31478-bib-0030] Zhang, M. , Lee, D.‐J. , Lillywhite, K. , & Tippetts, B. (2017). Automatic quality and moisture evaluations using evolution constructed features. Computers and Electronics in Agriculture, 135, 321–327. 10.1016/j.compag.2017.02.012

[fsn31478-bib-0031] Zheng, H. , & Lu, H. (2012). A least‐squares support vector machine (LS‐SVM) based on fractal analysis and CIELab parameters for the detection of browning degree on mango (*Mangifera* *indica* L.). Computers and Electronics in Agriculture, 83, 47–51. 10.1016/j.compag.2012.01.012

